# The organization and implementation of community-based education programs for health worker training institutions in Uganda

**DOI:** 10.1186/1472-698X-11-S1-S4

**Published:** 2011-03-09

**Authors:** Dan Kaye, Andrew Mwanika, Gilbert Burnham, Larry W Chang, Scovia N Mbalinda, Isaac Okullo, Rose C Nabirye, Wilson Muhwezi, Hussein Oria, Stephen Kijjambu, Lynn Atuyambe, Warren Aryeija

**Affiliations:** 1 School of Medicine, College of Health Sciences, Makerere University, Kampala, Uganda; 2 Department of Dentistry, School of Health Sciences, College of Health Sciences, Makerere University, Kampala, Uganda; 3 Department of Nursing, School of Health Science, College of Health Sciences, Makerere University, Kampala, Uganda; 4 Department of Pharmacy, School of Health Sciences, College of Health Sciences, Makerere University, Kampala, Uganda; 5 School of Public Health, College of Health Sciences, Makerere University, Kampala, Uganda; 6 Kabale University, Kabale, Uganda; 7 Johns Hopkins School of Public Health, Baltimore, Maryland, 21205, USA; 8 Johns Hopkins School of Medicine, Baltimore, Maryland, 21205, USA

## Abstract

**Background:**

Community-based education (CBE) is part of the training curriculum for most health workers in Uganda. Most programs have a stated purpose of strengthening clinical skills, medical knowledge, communication skills, community orientation of graduates, and encouragement of graduates to work in rural areas. This study was undertaken to assess the scope and nature of community-based education for various health worker cadres in Uganda.

**Methods:**

Curricula and other materials on CBE programs in Uganda were reviewed to assess nature, purpose, intended outcomes and evaluation methods used by CBE programs. In-depth and key informant interviews were conducted with people involved in managing CBE in twenty-two selected training institutions, as well as stakeholders from the community, Ministry of Health, Ministry of Education, civil society organizations and local government. Visits were made to selected sites where CBE training was conducted to assess infrastructure and learning resources being provided.

**Results:**

The CBE curriculum is implemented in the majority of health training institutions in Uganda. CBE is a core course in most health disciplines at various levels – certificate, diploma and degree and for a range of health professionals. The CBE curriculum is systematically planned and implemented with major similarities among institutions. Organization, delivery, managerial strategies, and evaluation methods are also largely similar. Strengths recognized included providing hands-on experience, knowledge and skills generation and the linking learners to the communities. Almost all CBE implementing institutions cited human resource, financial, and material constraints.

**Conclusions:**

The CBE curriculum is a widely used instructional model in Uganda for providing trainee health workers with the knowledge and skills relevant to meet community needs. Strategies to improve curricula and implementation concerns need further development. It is still uncertain whether this approach is increasing the number graduates seeking careers in rural health service, one of the stated program goals, an outcome which requires further study.

## Background

For more than two decades medical educators have used community-based medical education (CBE) programs to encourage the selection of careers in primary care serving rural populations. In developing countries, those in the Philippines, Nigeria, Nepal, South Africa, and Mali, have been described. In developed countries programs have been most actively promoted in Canada and Australia, but also in USA, and UK [1-10]. These programs are commonly designed around various blocks of clinical instruction, up to 16 weeks per year in rural areas, with some programs having their final medical school year almost entirely in rural locations [9]. Typically, contents include areas such as community diagnosis, health care delivery, family health, applied epidemiology, research methodology, and management skills for health services. The role of community engagement is seen as an important component to the success of rural clinical education [8].

Community-based education (CBE) has been part of training curriculum for most health workers in Uganda for some years. Stated goals are to promote the understanding of community issues for health workers and encourage careers providing primary health care to underserved rural areas. Although 13% of Uganda’s population lives in urban areas, 70% of doctors, 80% of pharmacists and 40% of nurses and midwives are based in urban areas [11,12]. On average, there is one physician and seven nurses or midwives per 100,000 population, both below the regional average [13]. Nationally, only about half of established positions for doctors are filled, and there is a high physician absenteeism rate [14]. These figures are undoubtedly worse in rural areas.

The Makerere School of Medicine introduced a Community-Based Education and Services (COBES) program in 2003 after an initial feasibility study in 2000, and at the same time a problem-based learning curriculum was added. All four medical schools in Uganda have followed with similar programs. For Makerere University College of Health Sciences (MakCHS), COBES is seen as an approach to foster interdisciplinary collaboration and self-directed learning through community placements in rural areas. The student groups include medicine, nursing, dentistry, pharmacy and radiology. The program is intended to instill in students the importance of developing community partnerships, engaging communities as a means to implement sustainable healthcare initiatives, and develop their skills and competence in accomplishing these goals. This exposure is intended to increase awareness of rural health problems and the conditions and lifestyles of health professionals in rural areas. While it does not guarantee immediate or consistent success in directing students into careers in rural practice it does present awareness of this option.

In the MakCHS COBES program students spend six weeks each year from years 1 to 5 in one of 41 rural areas working as a team in community and clinical activities. Students from the Makerere nursing, and allied health sciences training programs have participated with the medical students in COBES activities since shortly after the program began. The School of Public Health operates its own extensive community-based program for graduate students separate from COBES.

While many health professional training institutions in Uganda conduct some form of CBE, under a variety of names, the nature of this training has not been fully documented. There is little information on objectives, administration, available resources, and the challenges faced by these institutions. Documentation and sharing of this information, as well as potential solutions might not only improve the training of health professionals but also improve the recruitment, deployment and/or retention of health workers in rural areas.

As part of a Makerere University-Johns Hopkins University twinning project funded by the Bill and Melinda Gates Foundation, a better understanding of CBE programs was identified as a priority area in improving health worker training. This evaluation framework was developed to examine the organization and structure of programs as well as the outcome as indicated by client satisfaction (community, alumni and student) [15-17]

To gain a comprehensive picture of how CBE is provided in various programs in Uganda a survey was carried out of community rotations for 22 community-based programs. These programs were purposively selected from the 63 training programs identified as having a community-based educational component in Uganda. A stratified approach was used to represent various types of health worker training programs by location, academic program and level of training. Included were five degree, eight diploma and nine certificate awarding programs (Table 1).

**Table 1:** Institutions assessed for CBE

Institution	Degree Awarded	Management
Butabika School of Psychiatric Nursing	Diploma	Public-Government funded (admits private students)
Fort Portal School of Clinical Officers,	Diploma	Public-Government funded (admits private students)
Gulu School of Clinical Officers	Diploma	Public-Government funded (admits private students)
Chemequip Medical Laboratory Training School, Ishaka	Diploma, certificate	Private for profit
Gulu University Medical School	Degree	Public-Government funded (admits private students)
Ishaka School of Nursing and Midwifery	Diploma, certificate	Private not for profit
Jinja Medical Laboratory School	Diploma, certificate	Public-Government funded (admits private students)
Kagando School of Nursing and Midwifery	Diploma, certificate	Private not for profit
Kampala International University	Degree, Diploma	Private for profit
Kiwoko Medical Laboratory Training School	Diploma	Private not for profit
Kiwoko Nursing Training School (Enrolled and Comprehensive Nursing)	Diploma, certificate	Private not for profit
Lacor Medical Laboratory Assistants School	Diploma	Private not for profit
Lira Enrolled and Comprehensive Nursing School	Diploma, certificate	Public-Government funded (admits private students)
Masaka School of Comprehensive Nursing	Diploma, certificate	Public-Government funded (admits private students)
Mbale Clinical Officers' Training School,	Diploma	Public-Government funded (admits private students)
Mbarara University of Science and Technology	Degree	Public-Government funded (admits private students)
Ngora Comprehensive Nurses Training School	Diploma, certificate	Private not for profit
Nkozi University	Degree, diploma	Private not for profit
Rakai Community School of Nursing	Diploma, certificate	Private not for profit
Soroti School of Comprehensive Nursing	Diploma	Public-Government funded (admits private students)
Makerere University College of Health Sciences	Degree	Public-Government funded (admits private students)
Mbale School of Hygiene	Diploma, certificate	Public-Government funded (admits private students)

## Methods

Curricula, other training materials and reports were solicited from the 22 selected sites. From these a series of questionnaires were developed to assess multiple facets of CBE activities through quantitative and qualitative approaches. These covered choice of locations, selection of participants, supervision, learning activities, accommodation, extent of community participation, nature of the curricula, funding, reference materials available, and methods of trainee evaluation.

### Population and participant selection

Data were collected from October to November 2009. The programs evaluated represented institutions that offer degree, diploma or certificate level training, are public and private, and have rural and urban locations.

### Study design

This was a cross-sectional study involving the following data collection procedures. Triangulation was used to gain a comprehensive picture of how CBE functions across institutions. This included the following activities:

a) Review of available curricula and other CBE documents at the selected institutions to assess nature, purpose, intended outcomes and methods of assessment of training at the selected units

b) Visits to CBE sites to assess infrastructure and learning resources available.

c) In-depth interviews with CBE program leadership at the selected institutions.

### Variables

Program assessments included location of training, sponsoring organizations, key administrative personnel and their roles or responsibilities regarding community-based education, number of students rotating annually, number of supervising faculty and how the community-based training was financed. Information was collected on the site selection process, preparation for use by students, tutor selection and training, and the supervision and coordination of the program. The nature of the CBE curriculum, its goals and objectives and the intended outcomes were assessed. The learning process was reviewed for components that were reflective and experiential, self directed and for the nature of the feedback and mentorship of trainees. Finally, the assessment plan for trainees and any explicit process for curriculum evaluation were queried.

For service during the CBE attachment, the evaluation assessed the opportunities for reflective learning (creation of awareness and noting of critical incidents on which students reflect as they learn, eventually developing a learning plan); what activities were conducted by tutors, trainees and supervisors, to which sites students were attached, and how these sites were prepared for the trainees. Finally, the evaluation queried whether collaborative activities or community outreaches existed, and whether there was a student-led evaluation component attached to the community placements. The latter assessment looked for community diagnosis activities, the conduct of community assessments, collaborative assessments with community, participation in these assessments by CBE site tutors or supervisors, and the debriefing of the community after these activities.

Data were collected by faculty and research staff from Makerere University. The quantitative data were entered into EpiData and exported to SPSS for analysis. Qualitative data were analyzed using latent content analysis whereby themes were generated and categorized according to study questions, objectives and responses.

Ethical approval was received from MakCHS and the Uganda National Council for Science and Technology (UNCST). Informed consent was obtained from study participants and no personal data were recorded. The research was approved by the Institutional Review Board of the College of Health Sciences, Makerere University and received a waiver from Johns Hopkins University Bloomberg School of Public Health.

## Results

### Location, institution, and training site

There were six training institutions located in rural areas and 16 in urban areas. However, 18 of the CBE sites were rural and four were urban. The training institutions variously called CBE programs: field attachment, Primary Health Care field attachment, Community Health-Clinical Medicine, Community-Based Education, Research, Management and Service (COBERMS), Community-Based Education and Services (COBES), Community-based Health Education, Community Clerkship, Community Health – Primary Health Care, Health Promotion and Education, Hospital and Health Centre attachment, and Primary Health Care Practicum. All programs involved students working in health centers, and 17 of the 22 reviewed also included work in district or NGO hospitals. The number of students from each training institution rotating through community sites annually varied between 6 and 430, with half of the programs having less than 80 students going to field sites each year. Medical students spend six weeks per year at community sites in each of five years, and this is standard for all medical schools. Efforts are made to have medical students return to the same communities each year. Nursing students spend 4-8 weeks in community rotations, depending on their program.

### Administrative structure

The descriptions and administrative structure for CBE programs varied across institutions (Table 2). In some institutions a committees or a secretariat was responsible for program coordination, and in others it was a designated coordinator. Deans, tutors, and heads of department were the key personnel running the daily CBE activities. In 11 institutions, there was a coordinator who managed the overall day-to-day CBE activities, and another 11 institutions had a team in charge of CBE. The direct supervision and coordination of CBE program differed for the various institutions but these included institutional faculty, project coordinators, clinical instructors and hospital staff or clinic managers.

**Table 2:** Description of CBE at different institutions

Description	Nature of institutions	Administration of CBE
Field attachment to health facilities (hospitals) or established private laboratories	Laboratory training institutions (both diploma- and certificate-awarding)	Principal tutor, Deputy principal tutor
CBE being part of Primary Health Care	Clinical officer training institutions (Diploma-awarding)	Principal, Deputy principal tutor, Coordinator and Tutors,
COBERMS (community based education research management services)	Kampala International University (Degree-awarding institution)	Year coordinators Administrative Secretary, CBE Coordinator, COBERMS Under The Deans’ Office, COBERMS Committee,
Community-based education and service	Makerere University and Mbarara Universities (both degree-awarding)	COBES Chairperson COBES Committee COBES administrator COBES Supervisors COBES site tutors
Clinical attachment as part of community health	Certificate and diploma awarding nurses training institutions	Academic Registrar, Principal, Deputy principal, Coordinator and Tutors
Community clerkship (students learn in contexts outside their institutions)	All institutions	See specific institutions
Community health, primary health care, health promotion and education, hospital/health centre attachment	Clinical officer training institutions (Diploma awarding) All diploma awarding nurse-midwife training institutions	Principal, Deputy principal, Coordinator and Tutors. (Activities under the Department of Community Health, led by Heads of Department of Community Health and supported by Health Educators)
PHC practicum	Certificate awarding nurse-midwife training institutions	Heads of Department of Community Health, Health Educators.

All the training institutions surveyed had a budget for CBE. All reported inadequate financial resources to support the CBE activities as they were currently implemented. Inadequate funds limited the capacity of sites to provide activities such as student supervision, provision of internet connectivity and library facilities, provision of accommodation at CBE sites and facilitation of laboratories at field sites.

### Selection of sites and faculty

The type of CBE sites differed according to the programs, with some sites serving only as clinical or laboratory training sites, and others included community-based activities. Most sites were health centers. When new community or health facility sites were being selected, it was common for pre-visits to take place which involved not only clinic staff but visits to community leadership and often local government. In a few cases formal memorandum of understandings were signed. For one institution, students were encouraged to select their own sites. The following were criteria reported for selection ofsites: proximity and availability of health facilities, specific types of services offered such as maternal child health, mental health, the availability, and willingness of facility staffs to supervise students, and acceptance of students by community leadership.

Some academic institutions reported the reluctance or refusal of some health facilities to take CBE students on attachment. Some facilities already hosting students were concerned that increasing numbers of students would strain their capacity as well as that of the community in which many activities were conducted. As the number of health sciences schools and universities has increased in Uganda, the competition within and among schools for CBE training sites has become a concern. The number of well-run rural facilities with adequate staff and good access to study communities where students can be attached was felt by many interviewed to be limited in number. There were some instances in which a field site was shared between two training institutions, but tutors and supervisors were not shared. Among other academic institutions there was an intense competition for field training sites.

The roles and responsibilities of supervisors in CBE varied according to the sponsoring institution’s objectives as did trainee activities the different institutions (Table 3). For tutor selection and training, 18 of the institutions visited said that there was no formal selection and/or training of this vital group in CBE education. A detailed program for this was present in only four institutions visited. However, most tutors felt adequately prepared. CBE supervision and coordination differed from institution to institution. In some it was either a specific faculty member, a specified CBE project coordinator or a clinical instructor in charge of CBE. Some had a CBE coordinator working with a few faculty members or they had a specific department (e.g. Department of Public Health). In others the responsibility was borne by the principal tutors, a team of tutors, or staff in health facilities headed by the health facility managers. The numbers of supervising faculty varied across sites from 1 to 30, with half of training programs having six or less. Duties of the tutors included lectures, training in practical skills, supporting community outreach activities, assessment of student learning, and preparing reports.

**Table 3:** Main activities of site tutors and supervisors

CBE site tutors	Faculty (and supervising faculty)
Assessment of students' problems & supervision of student's activities	Assisting students in demonstrations, return demonstrations, improving the learning environment

Guidance, listening to student’s presentations, giving feedback to students	Checking on site tutors, learning problems and other trainee’s management work

Lectures, practical demonstrations, group discussions, follow-up, deploying students for fieldwork	Evaluation and assessment, teaching, corrective actions, feedback to students

Linking the health facility and community to the trainees, outreaches	Staying at community sites with students for on-site skills demonstration. (Kampala International University)

Monitoring students’ progress, teaching students	Observation, teaching, guiding, demonstration, marking, listening to students presentation

Observing trainees as they demonstrate skills and teach one another	Monitor performance of professional tasks and assess progress; Monitor student progress and welfare

Tutoring trainees and leading students in practical aspects of CBE	Management of trainees’ discipline
	Planning, follow-up of students, marking trainee’s reports
	Ensuring welfare of students in the field and reinforcing skills
	Signing student's log books, mentoring, counseling,

### CBE curricula

Table 4 shows the extent to which curricula are implemented as well as the instructional methods, student activities, and learning context used to ensure experiential and contextual learning. The majority of institutions had a CBE curriculum with clear goals, objectives and intended outcomes, but only nine had an evaluation plan. CBE was perceived as a subject in eight institutions, a course in eight institutions and a program in four institutions. Responses were not reported for two institutions. In all institutions, CBE involved a PHC practicum. Here trainees are attached to communities to appreciate health determinants and for community diagnosis. Other intended outcomes are acquisition of skills in creating community awareness on common diseases or conditions, disease prevention and health promotion; experiential learning in some cases including laboratory work, use of equipment and infection prevention.

**Table 4:** Assessment of CBE at the 22 institutions

Characteristic	Number (Percentage)
***Evaluation of the curriculum***	
There is a CBE curriculum	20 (90.9)
The curriculum have goals and objectives	18 (81.8)
The curriculum has clear intended CBE outcomes	16 (72.7)
The curriculum has an evaluation plan	9 (40.9)

***Implementation of CBE and resources available***	
There are community training sites	18 (81.8)
CBE site tutors are used	18 (81.8)
Learning takes place in the right context	18 (81.8)
Learning is self directed	15 (68.2)
There is immediate feedback to trainees	18 (81.8)
Libraries available	6 (27.2)

***Assessing for collaborative / social learning***	
Live in community or at a community hostel	8 (36.3)
Work on a group project	19 (86.4)
Work on individual project	11 (50.0)
Learn and participate in multidisciplinary teams	11 (50.0)
Write a group report	19 (86.4)
Write individual reports	17 (77.3)
Linked with alternative medical practitioners	10 (45.5)

***Instruction methods:***	
Lectures	20 (90.9)
Seminars	11 (50.0)
Workshops	8 (36.3)
Small groups	18 (81.8)
Learning problems	11 (50.0)
Case studies	10 (45.5)
Assignments	20 (90.9)
Skills demonstration	22 (100.0)

***The learning context:***	
Urban /periurban areas	15 (68.2)
District headquarters	7 (31.8)
Schools	15 (68.2)
Health centers or district hospitals	22 (100.0)
In homes	17 (77.3)
With non-government organizations	9 (40.9)

Table 5 shows the strategies to ensure experiential learning and attainment of desired competences: assessment competence, collaborative skills, knowledge, clinical skills, teamwork, and learning assessment methods. While students have prior training in assessment methodology, data analysis and report writing, only a few institutions require them to conduct some form of assessments. The assessment methods involved mainly continuous assessment with immediate feedback, oral reports, and written reports, but in only two institutions were marks given for the reports.

**Table 5:** Learning, research and assessment of learning

Characteristic	Number (Percentage)
***Strategies to promote understanding and learning***	
Structured group discussions	20 (90.9)
Peer feedback	16 (72.7)
Mentorship	15 (68.2)
Self-directed learning	21 (95.5)
Peer assessment	13 (59.1)
Portfolios	7 (31.8)
Community projects including community diagnosis	15 (68.2)

***Research during community-based training***	
There is a research component	10 (45.5)
There is operations research	5 (22.7)
Site tutors are involved (participate)	7 (31.8)
There is community diagnoses	9 (40.9)
Research is evaluated and marks are awarded	9 (40.9)
Research done as a follow up on community diagnosis	7 (31.8)
CBE sites/community receive feedback on findings	6 (27.2)
Trainees has prior knowledge: in research methodology	16 (72.7)
in data analysis	17 (77.3)
in report writing	18 (81.8)
Trainees have facilities for literature review:	
print media	13 (59.1)
electronic resources	7 (31.8)

***Assessment methods used***	
Observed activities, assessment with feedback	21 (95.5)
Log books	10 (45.5)
Oral reports (debriefing)	19 (86.4)
Peer assessment	17 (77.3)
Written reports	21 (95.5)
Progressive examinations at the institutions	17 (77.3)
A summative examination at the institutions	20 (90.9)
Presentation to a panel of examiners	6 (27.2)

### Student activities

Most students had some orientation or information to prepare them for field attachments in the form of lectures, small group work, seminars and workshops. Other learning methods mentioned included brainstorming, group discussions, drama, clerkships, outreaches, supervised patient care, role plays, self-study and conducting small projects. Indeed, tutors from 20 of the 22 facilities felt that their students were adequately prepared before coming to the field rotations. Some programs were primarily clinical or laboratory attachments, but there were 17 of the programs which included community outreach activities such as immunization programs, health promotion activities, and community diagnosis. Many programs followed up community assessments with health promotion or preventative medicine activities to address problems identified. At many field sites there was participation by a variety of local partners. Half of community programs reported involvement of traditional healers or traditional birth attendants. There were 15 programs that reported working with schools and nine with NGOs or other community organizations. Among NGOs were World Vision and TASO, others were established projects such as the JHU Rakai Project [18,19].

Assessment of learning was carried out through a variety of methods. All but one of the 22 sites stated they used observation, feedback and written reports to evaluate student work. Peer assessments, progressive and summative exams were also very common. Half of sites required students to keep logbooks of activities, and six sites required student presentations to a panel of examiners.

### The content of CBE training conducted

The content of CBE curricula was found to vary, ranging from topics on issues like health education, nutrition, mental health, antenatal care, immunization, water and sanitation, waste management, health facility management, and clinical or laboratory apprenticeship.. Facility-based activities involved running out-patient units, participating in ward rounds, and conducting maternal-child health activities such as antenatal care and immunizations. Community-based activities included conducting health education, community outreach and field researchwith community diagnosis. While trainees had prior training in assessment methodology, data analysis and report writing, not all students in field sites conducted some form of assessment or utilized evaluation methodology. The methods mainly involved continuous assessment giving immediate feedback, and oral and written reports. In only two institutions were marks given for the reports.

### Available resources to support CBE

Table 6 shows the available resources to support CBE. Most institutions had a budget for CBE, though all administrators thought this inadequate. There was no internet connectivity at 18 field sites. All facilities had consistent leadership at CBE sites, such as inspectors, in-charges of health units and political leaders, as well as facility staff and supervisors for the communities where trainees conducted outreach activities. Other resources were physical infrastructure with some CBE sites having hostels like those built by Mbarara University. At other sites transport to the CBE sites were provided, such as bus to take students to CBE sites or bicycles for use by trainees within the CBE sites and from the sites to the community. Some sites had television for student’s recreation.

**Table 6:** Resources at the 16 CBE sites visited to facilitate activities

Checklist of essential resources for CBE Site		Number present (%)
**Student Welfare**	Transport	15 (68.1)
	Accommodations	11 (50.0)
	Washing facilities	14 (63.6)
	Toilet facilities	15 (68.1)
	Food provision	13 (59.1)
	Cooking facilities	14 (63.6)
	Recreation/leisure	7 (31.8)
	Security	14 (63.6)
**Health Facility**	Community health activities	16 (100.0)
	Involvement of health workers in training	16 (100.0)
	Facilities for PBL	3 (13.6)
**Community**	Support from local leadership	15 (68.1)
	Involvement of traditional healers	10 (45.5)
	Involvement of partner organizations	7 (31.8)
	Involvement of community based organizations	13 (59.1)
	Community awareness of CBE	14 (63.6)
	Community acceptance	16 (100.0)
	Relevance of CBE activities	15 (68.1)
	Involvement of community in COBES	14 (63.6)
	General positive attitude towards CBE	15 (68.1)

### Scope of CBE implementation

There was continuous learning assessment in 18 institutions and summative assessment in 17. CBE promoted experiential learning at 20 sites, promoted service related learning in all 21, and promoted assessment methods at 13. For all institutions, most respondents felt that the curriculum objectives on CBE, the content, the instruction methods as well as learning assessment methods needed improvement. Other limitations identified were large number of students, limited funding, inadequate supervision, inadequate student welfare and inadequate learning materials while students are in the field.

### Student support

In many sites student accommodations were provided, but in some instances students had to pay for housing out of pocket. Transportation was a recurrent problem, both from the institution to the field site and then from the site to the community. Some sites had vehicles to reach the community sites, but in others, students had to walk or use bicycles. The lack of reference materials available to the students was noted at many sites.

### Perceived strengths and weaknesses of CBE training

Tutors and coordinators were asked about their perceptions of the strengths and weaknesses of their own CBE programs. Among strengths, tutors reported that programs had led to a progressively strengthening of collaboration among training institutions, health facilities and communities. Tutors believed that the promotion of service and education by CBE programs did have an impact on the health of the communities with which they worked. In several instances, they reported that the work of students was influencing local policy and practice. Program goals of improving student attitude and interest in health activities in rural areas they felt were being met Also noted as positive points were the opportunities for students to get hands-on practical experience, acquiring knowledge and skills complementing those from the classroom, and improved interaction with communities leaders and members. Other CBE strengths noted were the creation of good will with community and local leadership, and appreciation by the community of work of the students. Tutors felt the CBE rotations increased interest of students in rural practice.

Site staff we asked about their perception of the adequacy of the curriculum. The approach used by CBE curricula was rated high by staff at two institutions, adequate by faculty at 9 institutions and requiring further development at 11 institutions. The content of CBE curricula was felt to be of high quality by faculty and staff in three institutions, to be adequate in seven, and require further work in 12.

There were a number of weak areas identified by tutors and field staff. Many centered on the large number of students coming to field sites, inadequate supervision, insufficient staff with heavy responsibilities, and inadequate professional support to students in field sites. Some sites lacked sufficient patients, especially for the medical students. Inadequate financial support meant that many sites were forced to limit the length of CBE attachments. Family members of students are increasingly being asked to provide the financial support for transport and accommodation at some field sites, and some families lack the resources. The provision of computer facilities, training of site tutors, and the accommodation of students were frequently noted deficiencies. Poor motivation of tutors was a problem reported at a number of sites. Short of equipment at the health facility, insufficient staffing, and frequent drug stock-outs were felt to negatively affect the ability of students to learn essential skills, and lessened incentives for them to seek opportunities to work in rural areas in the future. Some tutors felt the length of rotation was too short for adequate learning.

Tutors also felt that students often lacked the opportunity or the ability to provide feedback to communities after conducting community assessments. This has made communities doubtful or skeptical about communities-learners partnerships. Several instances have occurred where students failed to get financial support from their training institutions to implement the planned, or promised community projects.

## Discussion

This first assessment of CBE activities in Uganda has provided a number of insights into the current status of programs, strengths and weaknesses, and evidence for future policy development. On the whole it has found the CBE approach to be a very widely accepted and appreciated component in all health worker training programs in Uganda, not just for medical doctors. Training programs for nursing, the allied health science and mental health workers all have community attachments. In all sites CBE has provided opportunities for experiential and contextual learning. The emphasis on learning in rural or disadvantaged environments adds an important diversity to the existing largely urban-based training. The opportunity to conduct community assessments and implement programs addressing problems identified is very widely appreciated. The linkages between training institutions and health facilities in the CBE activities is seen as an important link. Despite similar goals, there is wide variation in the concept and conduct of CBE at different health institutions. In spite of this, we believe the results of this study will help develop and refine policy to strengthen CBE programs in Uganda and elsewhere.

Although CBE is widely implemented in Uganda, there is no formal process to standardize programs, even among schools teaching similar disciplines. While some sites are multidisciplinary with students from several schools, other sites are aligned with specific courses such as training of laboratory technicians or clinical officers. Among faculty or site tutors there seemed to be an uncertainty about the adequacy or clarity of objectives for many programs. Without clear objectives, it is not surprising that evaluation of student performance was seen as deficient at some sites. A comprehensive evaluation strategy needs to include not only a thorough evaluation of learners, but also an assessment of the instructional design itself [20].

This study found that many training institutions reported difficulties with finding sufficient well run PHC facilities in rural Uganda that can serve as effective training centers. Drug stock-outs and the shortage of equipment and supplies were noted in the training facilities. The shortage of health facility staff limits the effective supervision that can be given to students. Many medical officer positions remain unfilled. High migration and absenteeism makes the situation worse [14]. Facilities struggling with these challenges are hardly role models for attracting persons into rural or PHC practices. Across CBE programs there were shortages in tutors, and difficulties with administration and implementation. Although community programs are at the core of most CBE programs, implementing these in a thorough and consistent manner is often difficult.

Based on the findings of this study there are a number of critical issues that need addressing include streamlining the implementation of curricula, optimizing available resources at the CBE sites or in the community, and strengthening the infrastructure for training and welfare (such as internet access). Provision of telemedicine equipment at CBE sites for both learning and service would encourage retention of health workers in rural areas. Considering the multi-factorial nature of CBE constraints, efforts to address them requires crucial partnerships between the health professional training institutions, the ministry of health, the local government authorities, the communities and other players in delivery of healthcare, such as NGOs. Such partnership may increase resources to improve the infrastructure at CBE sites, provide accommodation of trainees or transport to communities, finance the administration of CBE activities or provide social amenities for the welfare of the trainees at site tutors.

For developing a strong rural doctor workforce, 4-6 week annual rural rotations may not be the most effective approach, though it does build some community awareness. Longer rural clinical rotations were better at promoting rural service among doctors in Australia [21]. Several tutors at the sites visited in this study were of the same opinion. The Australian rural practice programs distribute much of the clinical training to field sites during the final years of service has been an effective approach [22]. In Illinois, US, a parallel program for rural practice placed medical students in the rural areas for the majority of their time and this showed a high success rate [8]. Another successful strategy for building a rural workforce has been the selective recruiting of students from rural areas, as well as early screening for motivational factors [23].

Now that CBE programs in Uganda have been running for a number of years, a serious assessment of outcomes is needed. CBE programs have proved to be expensive, with all sites reporting financial problems. There may be less expensive approaches to develop familiarization with rural health needs, which could allow some funding to be redirected to more intensive programs to build a dedicated cadre of health workers for rural or neglected-areas from among a subset of students.

This study has multiple potential limitations. The purposive sampling may have excluded sites where experiences were considerably different. Local factors varied among training sites. This paper focused mainly on structural and organizational perspectives. Perceptions of students, alumni and the community about the nature and adequacy of programs are reported elsewhere, but add light to many issues identified here. The methods used were unable to assess if the number of graduates seeking careers in rural areas had actually increased as a result of this substantial investment in CBE, and this needs further study.

## Conclusions

Community Based Education is widely implemented by academic programs training health workers in Uganda Even with the diverse nature of CBE programs, all face similar challenges and constraints related to the financing, administration and CBE evaluation. Experiential and contextual learning, are a clear benefit to trainees, and CBE provides a service health facilities as well as to communities. These CBE programs may not independently be associated with rural practice choices; however they are important co-factors providing contextual and experiential training experiences. Financial limitations are present in all programs. Alternative approaches may be required to meet goals of expanding the rural health workforce in Uganda. Future studies could comprehensively assess the impact of CBE in many areas such as understanding community need, development of specified competences and choosing careers in underserved areas. Other areas to be examined include how CBE programs could build interdisciplinary and inter-institutional collaboration, and community-campus partnerships to promote sustainable healthcare initiatives.

## List of abbreviations used

CBE: Community-based education; COBES: Community-Based Education and Services; MakCHS: Makerere University College of Health Sciences; COBERMS: Community-Based Education, Research, Management and Service.

## Competing interests

The authors declare that they have no competing interests.

## Authors' contributions

DK, AM, SM, IO, RCN, WM, and HO participated in the conception and design of the study, participated in the data collection and helped in the analysis, and drafting the manuscript. GB, WA, LC, and KS participated in the conception and design of the study and the data analysis and helped draft the manuscript. LA participated in the conception and design of the study and the data analysis. DK, AM, GB, and WA took major roles in the writing of the manuscript. All authors read and approved the final manuscript.
